# Stop GnRH-Agonist Combined With Multiple-Dose GnRH-Antagonist Protocol for Patients With “Genuine” Poor Response Undergoing Controlled Ovarian Hyperstimulation for IVF

**DOI:** 10.3389/fendo.2020.00182

**Published:** 2020-05-22

**Authors:** Raoul Orvieto, Michal Kirshenbaum, Valentina Galiano, Tal Elkan-Miller, Eran Zilberberg, Jigal Haas, Ravit Nahum

**Affiliations:** ^1^Infertility and IVF Unit, Department of Obstetrics and Gynecology, Chaim Sheba Medical Center, Tel Hashomer, Affiliated to the Sackler Faculty of Medicine, Tel Aviv University, Tel Aviv, Israel; ^2^Sackler Faculty of Medicine, Tel-Aviv University, Tel Aviv, Israel

**Keywords:** poor responders, COH, Bologna criteria, stop protocol, GnRH-antagonist

## Abstract

**Objective:** To examine whether the Stop GnRH-agonist combined with multiple-dose GnRH-antagonist protocol may improve conventional IVF/intracytoplasmic sperm injection (ICSI) cycle in poor ovarian response (POR) patients.

**Design:** Cohort historical, proof of concept study.

**Setting:** Tertiary, University affiliated Medical Center.

**Patient(s):** Thirty POR patients, defined according to the Bologna criteria, who underwent a subsequent Stop GnRH-agonist combined with multiple-dose GnRH-antagonist controlled ovarian hyperstimulation (COH) protocol, within 3 months of the previous failed conventional IVF/ICSI cycle, were included. For the purposes of this study, we eliminated a bias in this selection by including only “genuine” poor responder patients, defined as those who yielded up to 3 oocytes following COH with a minimal gonadotropin daily dose of 300 IU.

**Main Outcome Measure(s):** Number of oocytes retrieved, number of top-quality embryos, COH variables.

**Result(s):** The Stop GnRH-agonist combined with multiple-dose GnRH-antagonist COH protocol revealed significantly higher numbers of follicles >13 mm on the day of hCG administration, higher numbers of oocytes retrieved, and top-quality embryos (TQE) with an acceptable clinical pregnancy rate (16.6%). Moreover, as expected, patients undergoing the Stop GnRH-agonist combined with multiple-dose GnRH-antagonist COH protocol required significantly higher doses and a longer duration of gonadotropins stimulation.

**Conclusion(s):** The combined Stop GnRH-ag/GnRH-ant COH protocol is a valuable tool in the armamentarium for treating “genuine” poor ovarian responders. Further, large prospective studies are needed to elucidate its role in POR and to characterize the appropriate patients subgroup (before initiating ovarian stimulation) that may benefit from the combined Stop GnRH-ag/GnRH-ant COH protocol.

## Introduction

Controlled ovarian hyperstimulation (COH) is a crucial step in the success of *in vitro* fertilization-embryo transfer (IVF-ET), enabling the recruitment of multiple oocytes and subsequently, the vitrification of all surplus embryos (1). However, due to the extreme heterogeneity in ovarian response to COH in some patients, referred to as “low/poor-responders,” COH may only yield a few follicles, if any (2).

Until 2011, there was no one single definition for patients with poor ovarian response, though the most accepted criterion was a decreased response to COH, which, in IVF cycles, correlates to the reduced quantity of oocytes retrieved. The controversy surrounding the diagnosis of patients with poor ovarian response (POR) to ovarian stimulation resulted in a systematic standardization of the definition by the European society of Human Reproduction and Endocrinology (ESHRE), known as the Bologna criteria. According to the Bologna criteria, in order to define POR, “at least two of the following three features must be present: (i) Advanced maternal age (≥40 years) or any other risk factor for POR; (ii) A previous POR (≤3 oocytes with a conventional stimulation protocol); and (iii) An abnormal ovarian reserve test (3). In the absence of advanced maternal age or abnormal ovarian reserve tests, two previous maximal stimulation attempts with POR are sufficient to define a patient as a poor responder.”

Several treatment strategies are offered to patients with POR to COH. These include reducing or stopping the dose of GnRH-agonist (GnRH-ag), the ultrashort, short and microdose GnRH-ag (“flare” protocols), the use of GnRH-antagonist (GnRH-ant), the combined ultrashort GnRH-ag with the multiple-dose GnRH-ant, the co-administration of letrozole, the modified natural-IVF cycle (2, 4–8), or the use of different doses and types of gonadotropin preparations (9, 10). Nevertheless, despite the multiplicity of strategies, no clear conclusion has been established on which regimen would be the ideal COH protocol for patients defined as POR (11).

In 1998, Faber et al. were the first to introduce the Stop protocol aiming to improve treatment outcome in patients with POR. The Stop protocol combines down-regulation with GnRH-ag starting at the luteal phase, cessation of GnRH-ag therapy with the onset of menstruation and high-dose gonadotropin administration. This short-term ovarian suppression, which begun in the luteal phase and discontinued with the onset of menses, followed by a high-dose stimulation with gonadotropins, was demonstrated to yield favorable pregnancy results in low responders (12). Although promising, a Cochrane review by Maheshwari et al. assessing the most effective GnRH-ag protocol as an adjuvant to gonadotropins in ART cycles, could not demonstrate any evidence of a difference in any of the outcome measures for continuation vs. stopping of GnRH-ag at the beginning of stimulation and follicular vs. luteal start of GnRH-ag (13).

Several years ago, our group demonstrated that combining the ultrashort flare GnRH-ag and GnRH-ant protocols in POR patients, who previously failed several IVF treatments cycles, yielded a 14.3% clinical pregnancy rate (7). This protocol, “which combines the benefit of the stimulatory effect of GnRH-ag flare on endogenous FSH with the benefit of immediate LH suppression of the GnRH antagonist,” was therefore suggested as a valuable new tool for treating poor responders.

Based on the valuable addition of the ultrashort flare GnRH-ag combined with GnRH-ant to the COH protocols armamentarium (14), in the Chaim Sheba Medical Center, we started offering POR patients the combined Stop GnRH-ag with multiple-dose GnRH-ant protocol. In the present study, we sought to examine the role of Stop GnRH-ag combined with multiple-dose GnRH-ant in POR patients undergoing conventional IVF/intracytoplasmic sperm injection (ICSI) cycle. Assessing a new potentially promising treatment protocol will aid both fertility specialists' counseling and POR patients in adjusting their appropriate treatment strategy.

## Patients and Methods

We reviewed the computerized files of all consecutive women admitted to our IVF unit at the Chaim Sheba Medical Centre between January and November 2019. Inclusion criteria included patients with POR to conventional multiple-dose GnRH-antagonist IVF/ICSI cycles, defined according to the Bologna criteria (3), who underwent a subsequent COH using the combined Stop GnRH-ag with multiple-dose GnRH-ant protocol within 3 months of the previous *failed* conventional IVF/ICSI cycle. By only including a subgroup of “genuine” poor responder patients, those who fulfilled 2 out of 3 Bologna criteria and yielded up to 3 oocytes following COH with a minimal gonadotropin daily dose of 300 FSH IU, we eliminated potential selection bias. The study was approved by the institutional research ethics board of Sheba Medical Center.

In the initial conventional COH, gonadotropins were started on day 2–3 of the menstrual cycle (corresponding to stimulation day 1) in variable doses, with a minimal daily dose of 300 IU, depending on the patient's age and/or ovarian response in previous cycles. The continuing dose was adjusted according to serum E2 levels and vaginal ultrasound measurements of follicular diameter obtained every 2 or 3 days. GnRH-antagonist treatment (0.25 mg/day, Cetrorelix, Cetrotide, Serono International SR, Geneva, Switzerland or Orgalutran; NV Organon, Oss, The Netherlands) was started when a follicle reached 13 mm and/or E2 levels exceeded 400 pg/mL.

In the combined Stop GnRH-ag with multiple-dose GnRH-ant protocol, patients received triptorelin (Lapidot, Netanya, Israel) 0.1 mg/day, started in the midluteal phase and discontinued with the onset of menses and after confirmation of down-regulation by serum E2 levels and vaginal ultrasound measurements. Gonadotropins were initiated after two wash-out days, with maximal doses. Once the leading follicle had reached a size of 13 mm, and/or E2 levels exceeded 400 pg/mL, co-treatment with the GnRH antagonist 0.25 mg/day was initiated and continued up to, and including, the day of HCG administration.

Routine IVF or ICSI was performed, as appropriate. All patients received luteal support with progesterone. Embryos classification was based on the individual embryo scoring parameters according to pre-established definitions (15). A top-quality embryo (TQE) was defined as three or more blastomeres on day 2 and seven or more blastomeres on day 3, equally-sized blastomeres and <20% fragmentation. All other characteristics defined poor embryo quality.

Data on patient age and infertility-treatment-related variables were collected from the computerized clinical files. Outcome was assessed in terms of COH characteristics, cancellation rates, amount of gonadotropin required to COH, duration of stimulation, number of retrieved oocytes, number of TQE, number of embryos transferred, and pregnancy rates and compared between the previous conventional (Conventional-group) and the combined Stop GnRH-ag with multiple-dose GnRH-ant IVF/ICSI cycles.

Results are presented as means ± standard deviations. Comparison of continuous variables between the two groups was conducted using a Mann–Whitney *U* test or student *t*-test, as appropriate. Chi-square or a Fisher exact test were used for comparison of categorical variables. Significance was accepted at a probability value of <0.05.

## Results

Thirty “genuine” poor responder patients (age 37.4 ± 7.8 years) during a conventional IVF/ICSI cycle, who underwent a subsequent combined Stop GnRH-ag with multiple-dose GnRH-ant cycle, were evaluated. The clinical characteristics of the IVF cycles in the two study groups are shown in (Table 1).

**Table 1:** Clinical characteristics of the IVF cycles in the two study groups.

	**Control cycles**	**Study cycles**	***p*-values**
Number of cycles	30	30	
Cancellation rate (%)	56.7%	20.0%	0.002
Total dose of gonadotropin used (IU)	3,842 ± 1,702	5,372 ± 1,572	0.001
Length of stimulation (days)	8.4 ± 2.1	10.7 ± 2.8	0.001
Peak E2 levels on day of hCG administration (pmol/L)	1,841 ± 1,580	3,033 ± 2,003	0.01
Number of follicles >13 mm on day of hCG administration (range)	1.76 ± 1.13 (0–5)	3.53 ± 1.90 (1–9)	0.001
Number of oocytes retrieved (range)	1.33 ± 1.12 (0–3)	3.93 ± 2.91 (0–10)	0.001
Number of MII oocytes (range)	1.2 ± 1.06 (0–3)	3.43 ± 2.71 (0–9)	0.001
Number of TQE (range)	0.53 ± 0.73 (0–3)	1.65 ± 1.4 (0–4)	0.001
Number of embryos transferred (range)	0.53 ± 0.68 (0–2)	1.13 ± 0.77 (0–3)	0.001

As expected, the conventional IVF/ICSI cycles preceding the combined Stop GnRH-ag with multiple-dose GnRH-ant cycles were characterized by a significantly shorter COH (8.4 ± 2.1 vs. 10.7 ± 2.8, *p* < 0.001, respectively) and significantly lower requirement of gonadotropin doses (3,842 ± 1,702 vs. 5,372 ± 1,572, *p* < 0.001, respectively). Patients undergoing the combined Stop GnRH-ag with multiple-dose GnRH-ant cycles achieved significantly higher peak estradiol levels compared to those in the conventional cycles (3,033 ± 2,003 vs. 1,841 ± 1,580, *p* < 0.001, respectively) and higher numbers of follicles >13 mm in diameter on the day of triggering final follicular maturation (3.53 ± 1.90 vs. 1.76 ± 1.13, *p* < 0.001, respectively). Moreover, other COH outcomes were improved in the combined Stop GnRH-ag with multiple-dose GnRH-ant cycles compared to the conventional cycles, such as the number of oocytes retrieved (3.93 ± 2.91 vs. 1.33 ± 1.12, *p* < 0.001, respectively), MII oocytes (3.43 ± 2.69 vs. 1.08 ± 0.99, *p* < 0.001, respectively), TQE (1.6 ± 1.40 vs. 0.53 ± 0.73, *p* < 0.01, respectively) and the number of embryos transferred (1.13 ± 0.77 vs. 0.53 ± 0.77, *p* < 0.001, respectively) (Table 1).

Cancellation rates were 56.7% in the preceding conventional IVF/ICSI cycles, as compared to 20.0% in the combined Stop GnRH-ag with multiple-dose GnRH-ant cycles (*p* < 0.002). Of the six patients canceled in the combined Stop GnRH-ag with multiple-dose GnRH-ant cycles, five were also canceled in the previous conventional cycle. No patients conceived following the previous conventional IVF/ICSI cycles, while five pregnancies (16.6%) were recorded in the Stop GnRH-ag with multiple-dose GnRH-ant group.

## Discussion

In the present cohort historical, proof of concept study of “genuine” POR patients, according to the Bologna criteria, who achieved ≤3 oocytes following COH with conventional IVF/ICSI, the combined Stop GnRH-ag with multiple-dose GnRH-ant cycle provided significantly higher numbers of oocytes retrieved, as well as higher numbers of embryos transferred, as compared to their previous IVF attempt. Five clinical pregnancies (pregnancy rate, 16.6%) were recorded. However, it should be emphasized that this reasonable pregnancy rate in the combined Stop GnRH-ag with multiple-dose GnRH-ant cycle is biased due to the study design, which offered this protocol to poor-responder patients who had failed a previous IVF attempt.

When considering the additional benefit of increasing the oocyte yield in POR, it has been demonstrated in all age groups, that the retrieval of merely one more oocyte (2 instead of 3 oocytes) increases the cumulative live birth rate (LBR) per cycle by ~25% (16). Moreover, a retrospective study by Drakopoulos et al. (17), evaluating the cumulative LBR deriving from one stimulation cycle (following fresh and frozen-thawed transfers), has demonstrated that low response patient (1–3 oocytes) achieved a significantly lower cumulative LBR compared to suboptimal response patient (4–9 oocytes). Therefore, the additional two oocytes retrieved and one TQE in the present study of genuine POR undergoing the combined Stop GnRH-ag with multiple-dose GnRH-ant cycle, may explain the observed improvement in the IVF outcome with a reasonable live birth rate.

The rationale behind the sequential treatment of the combined Stop GnRH-ag with multiple-dose GnRH-ant protocol stems from the advantages of its components. The long GnRH-ag protocol pretreatment results in better synchronized response and a scheduled cycle (18, 19). Moreover, since continuing the GnRH-ag during COH is often associated with a significant increase in the number of gonadotropin ampoules required for achieving adequate follicular development, its cessation might improve ovarian response and avoid the need of increasing the gonadotropin daily dose. GnRH-ag causes suppression of pituitary LH secretion for as long as 10 days after the last dose of the agonist (20), which, together with the immediate LH suppression provided by the GnRH-ant, will eliminate premature LH surge and may improve the quality of the embryos generated. In POR, GnRH-ant down-regulation has an additional advantage in that final oocyte maturation may be triggered by GnRH agonist together with hCG (Double trigger), with an improved IVF outcome (21).

In our previous observation in this subgroup of “genuine” poor responders, we demonstrated that clinical pregnancy was observed in 4% in their subsequent IVF cycle using conventional COH (8). Moreover, according to a recently published study by our group, the reported live birth rates per cycle for poor responder patients using a daily gonadotropin dose of 450 IU resulted in 7.7% (9). These figures are in accordance with the present study, reflecting a reasonable IVF outcome using the combined Stop GnRH-ag with multiple-dose GnRH-ant protocol in this frustrating group of “genuine” POR.

A limitation of our analysis is its retrospective design and the small sample size. However, based on our patients' selection process, we enrolled only consecutive patients fulfilling the inclusion criteria, therefore, considerably decreasing the likelihood of selection bias. In addition, the combined Stop GnRH-ag with multiple-dose GnRH-ant cycle outcomes were compared to the previous COH-IVF of the same patients, thus aiming to eliminate any matching hurdles.

In conclusion, we chose to concentrate on a specific population among all POR (according to the Bologna criteria) with ≤3 oocytes following conventional COH for IVF with a high (>300 IU) daily dose gonadotropins, because these patients are most challenging. In the present study, the combined Stop GnRH-ag/GnRH-ant COH protocol was demonstrated to be a valuable tool in the armamentarium for treating “genuine” poor ovarian responders. Further, large prospective studies are needed to identify the specific characteristics of POR patients (before initiating ovarian stimulation) who may benefit from the combined Stop GnRH-ag/GnRH-ant COH protocol.

## Data Availability Statement

The raw data supporting the conclusions of this article will be made available by the authors, without undue reservation.

## Ethics Statement

The studies involving human participants were reviewed and approved by IRB Sheba Medical Center. Written informed consent for participation was not required for this study in accordance with the national legislation and the institutional requirements.

## Author Contributions

RO was the principal investigator, designed the study, performed the statistical evaluations, assisted in writing the paper, and edited it in all its revisions. MK, TE-M, EZ, and JH participated in designing the study, assisted in writing the paper and edited it, proof read the paper, and took part in discussions regarding the results. VG retrieved the data, assisted in writing the paper and edited it, proof read the paper, and took part in discussions regarding the results. RN participated in designing the study, retrieved the data, assisted in writing the paper, and edited it in all its revisions. All authors read and approved the final manuscript.

## Conflict of Interest

The authors declare that the research was conducted in the absence of any commercial or financial relationships that could be construed as a potential conflict of interest.

## Abbreviations

COH: Controlled ovarian hyperstimulation
GnRH-ag: GnRH-agonist
GnRH-ant: Gonadotropin-releasing hormone-antagonist
ICSI: intracytoplasmic sperm injection
IVF-ET: *in vitro* fertilization-embryo transfer
POR: poor ovarian response
TQE: top-quality embryos.
